# The Acrosomal Status of Density Purified Spermatozoa Differentiates Men from Couples in IVF and ICSI Treatment and Is Associated with Fecundity

**DOI:** 10.3390/jcm9082327

**Published:** 2020-07-22

**Authors:** Pernille Badsberg Norup, Dorte L. Egeberg Palme, Morten R. Petersen, Katharina M. Main, Kristian Almstrup

**Affiliations:** 1 Department of Growth and Reproduction, Rigshospitalet, University of Copenhagen, DK-2100 Copenhagen, Denmark; pernille.badsberg.norup.01@regionh.dk (P.B.N.); katharina.main@regionh.dk (K.M.M.); 2 International Center for Research and Research Training in Endocrine Disruption of Male Reproduction and Child Health (EDMaRC), Rigshospitalet, University of Copenhagen, DK-2100 Copenhagen, Denmark; 3 European Sperm Bank ApS, DK-2200 Copenhagen, Denmark; deg@europeanspermbank.com; 4 The Fertility Clinic, Rigshospitalet, University of Copenhagen, DK-2100 Copenhagen, Denmark; morten.roenn.petersen@regionh.dk

**Keywords:** fertility treatment, acrosome, sperm, infertility, male factor, semen analysis

## Abstract

The acrosome of the spermatozoa is required for fertilization and in the raw ejaculate the percentage of viable acrosome-intact spermatozoa, the acrosomal status, is higher among men with good semen quality. Here we investigated if the acrosomal status of the processed semen preparations used at a fertility clinic can also be informative and whether it is associated with fecundity. The acrosomal status was measured by image cytometry on purified semen samples from couples during in vitro fertilization (IVF) (*n* = 99) and intracytoplasmic sperm injection (ICSI) (*n* = 107) treatment. Purified frozen-thawed donor samples were also analyzed (*n* = 199). In purified semen preparations the acrosomal status was significantly higher among sperm donors (*p* = 5.3 × 10^−8^) and men from IVF couples (*p* = 2.2 × 10^−5^) when compared to men from ICSI couples. A significant difference was also found between female, male and mixed factor infertility (*p* = 0.003). No association with lifestyle factors was found. In frozen-thawed donor samples, a significant positive (r = 0.16, *p* = 0.025) association with the number of pregnancies per sold straw was observed together with an area under the curve of 75.3%, when comparing the top and bottom deciles. Our results indicate that the acrosomal status may be a valuable parameter for personalizing fertility treatments and might be a good predictor of pregnancy success among normozoospermic men.

## 1. Introduction

The number of couples seeking help for infertility is increasing worldwide [1,2,3]. In 2018, approximately 1 out of 10 Danish children was born after fertility treatment [4]. In approximately half of the infertile couples, low semen quality seems to be a critical factor [5]. Consequently, evaluation of semen quality is important in the clinical work-up of infertile couples and contributes to the clinical decision tree for choosing the type of treatment, i.e., intrauterine insemination (IUI), in vitro fertilization (IVF) or intracytoplasmic sperm injection (ICSI). IUI is the least invasive treatment and can be used if the male partner presents with good semen quality. On the contrary, ICSI treatment is often the choice when the semen quality is poor or if fertilization fails during IVF. For some couples, semen quality is, however, too poor and donation of sperm is needed to obtain pregnancy. Thus, both the use of assisted reproduction and the demand for donor sperm have increased in the past decades [4,6]. However, despite increased use of donor sperm and significant advances in assisted reproductive techniques (ART), especially ICSI, many couples remain childless. The Danish fertility clinics reported in 2018 that only 22% of all ART treatments (IVF and ICSI) and 11.6% of the IUI treatments resulted in a live birth [4].

Semen quality is usually evaluated manually by determination of classical semen parameters like sperm concentration, morphology and motility [7]. However, the predictive value for fertility is limited if the semen quality is not extremely affected [8]. Other factors, such as the integrity of the sperm DNA, chemical and immunological factors in the seminal fluid, the ability to respond to stimuli in the female tract and multiple unknown factors play a role in fertilization [9,10,11]. Only human spermatozoa with an intact acrosome that are capable of undergoing acrosome reaction can penetrate the zona pellucida and fertilize the oocyte [12]. Thus, an important parameter could be the ability of the spermatozoa to undergo acrosomal exocytosis at the right time and place. Lectins like Pisum Sativum Agglutinin (PSA) bind selectively to the acrosomal matrix and can be used to test for the presence of the acrosome [13,14]. Traditionally, assays to investigate the functionality of the acrosome involve quite laborious manual procedures, and they have not been used in the clinic or in larger clinical studies. Recently, we have developed an assay based on image cytometry, which allows automatization and easy determination of the percentage of viable acrosome-intact spermatozoa in an ejaculate, an indicator of the acrosomal status [15,16]. This assay exploits the advantage of PSA coupled to fluorescein isothiocyanate (FITC) to flow via membrane pores into the acrosomal compartment of reacting acrosomes and to stabilize the acrosomal matrix allowing easy detection of spermatozoa undergoing the acrosome reaction [17]. Addition of Propidium Iodide (PI) further allows discrimination between viable and non-viable spermatozoa [15], whereby dead or dying spermatozoa with destabilized membranes can be masked. Hence, when PI and PSA are used in combination, the percentage of viable acrosome-intact spermatozoa - the acrosomal status - can be determined.

In the fertility clinic, semen samples are purified in order to obtain a fraction of concentrated, high-quality spermatozoa free from round cells and seminal fluid. Spermatozoa with abnormal morphology are, at least to some extent, excluded in the purified fraction [18,19]. The question remains whether this purification also eliminates e.g. the observed difference in the acrosomal status in raw ejaculates of men from couples in IVF and ICSI treatment [15].

The objective of this study was to investigate the acrosomal status of the spermatozoa directly used in fertility treatment, i.e., density gradient purified semen preparations from infertile couples and purified-frozen-thawed preparations from sperm donors. We aim to test if differences in the acrosomal status exist between men from couples in IVF and ICSI treatment and between men from couples with different causes of infertility. We further aimed to test whether the acrosomal status could be associated with lifestyle factors of the infertile couples and fecundity among semen donors.

## 2. Experimental Section

### 2.1. Study Population

To investigate the acrosomal status among infertile couples a total of 240 HIV- and hepatitis-negative semen samples from infertile couples in fertility treatment were obtained on a random basis over a period of 1.5 years. Only samples with a surplus of spermatozoa after treatment could be included. Thirty-four semen samples were excluded for quality reasons (too few cells, see below). The remaining semen samples were from couples treated with IVF (*n* = 99) and ICSI (*n* = 107). At the Fertility Clinic, Rigshospitalet, couples were assigned to IVF or ICSI based on standardized criteria, i.e., couples with no tubal factor infertility and with a minimum of two million progressive motile spermatozoa after purification are referred to IUI; in cases of tubal factor infertility, or one or more failed IUI cycles, couples are referred to IVF. Couples with <two million progressive motile spermatozoa after purification or one or more failed IVF cycles are referred to ICSI [20]. Cycle information including treatment type, treatment number, semen analysis, aspirated oocytes, fertilization and pregnancy details was extracted on a per-cycle-basis from the national clinical database: DMDC Journal (Dansk Medicinsk Datacenter ApS). Furthermore, information on the consumption of alcohol, smoking, body mass index (BMI), and infertility diagnosis was extracted for both partners. 

To investigate the acrosomal status among semen donors, 209 frozen-thawed and randomly selected samples from 146 sperm donors from the European Sperm Bank were included. Data regarding sold straws, the number of reported pregnancies, BMI and age were obtained from the European Sperm Bank databases. The number of reported pregnancies per sold straw was used as a measure of fecundity. Only data on the number of pregnancies and the number of straws sold per donor was available and no information was available about the kind of treatment and the women being treated. To eliminate bias driven by single female factors, a lower cut off of 25 sold straws per donor was chosen because this assumed that the donor had been used in the treatment of more than three different women. Ten acrosomal status measurements were excluded due to fewer than 25 sold straws. The remaining 199 donor semen samples were from 138 unique donors. As freezing and thawing affect the viability of spermatozoa and, hence, the acrosomal status, 48 randomly selected density gradient purified samples from donors also had acrosomal status measured before freezing to allow the direct comparison of acrosomal status between sperm donors and samples from men from infertile couples.

To investigate the variation of acrosomal status over time, a subset of donors (*n* = 8) had the acrosomal status of the raw samples measured repetitively (range: 3–24 times) over a period of 11 months.

### 2.2. Collection and Processing of Semen Samples

Semen samples were produced by masturbation and ejaculated into clean, wide-mouthed plastic containers after at least 48 h of abstinence. Samples from men from infertile couples were produced at home and delivered within 2 h after ejaculation at the fertility clinic in the morning of the day in which oocyte aspiration for the partner was planned. Samples from sperm donors were produced at the European Sperm Bank. The raw samples were evaluated according to WHO criteria for ejaculate volume, sperm concentration and progressive motility by standard procedures at both locations. Evaluations were performed in duplicates. Semen samples from men from infertile couples were purified by density gradient centrifugation (PureCeption, SAGE Media, Trumbull, CT, USA), washed in Quinn’s Sperm Washing Medium (CooperSurgical, Maaloev, Denmark) three times (350× *g*) and diluted to 3 mill/mL progressive motile spermatozoa for IVF treatment and 1 mill/mL progressive motile spermatozoa for ICSI treatment. After completed IVF or ICSI procedures, the remaining processed samples were collected and acrosomal status measured (see below). Semen samples from sperm donors were processed by density gradient centrifugation (Sil-Select, FertiPro, Beernem, Belgium) and washed in FertiCult Flush (FertiPro, Beernem, Belgium). After processing the samples were diluted in freeze medium (SpermFreeze SSP, FertiPro, Beernem, Belgium) and analyzed before cryopreservation. After freezing, a control was thawed and the number of progressively motile spermatozoa cells per milliliter was counted before the acrosomal status was measured (see below). 

### 2.3. Measurement of Acrosomal Status

The assay for the acrosomal status (the percentage of viable acrosome-intact spermatozoa) is described in detail elsewhere [15,16]. A few modifications to the published protocol were made. In brief, the purified semen samples were washed twice in Dulbecco’s phosphate-buffered saline (PBS, ThermoFisher Scientific, Roskilde, Denmark) (700× *g*, 10 min). The spermatozoa pellet was resuspended in a staining solution containing (final concentrations): 5 μg/mL fluorescein isothiocyanate conjugated Pisum sativum agglutinin (FITC-PSA, Sigma-Aldrich, Soeborg, Denmark), 0.5 μg/mL propidium iodide (PI, ChemoMetec A/S, Alleroed, Denmark), and 10 μg/mL Hoechst-33342 (H342, ChemoMetec, Alleroed, Denmark) in PBS and incubated for 30 min at 37 °C. A thorough mix was made by pipetting, a 50 μL aliquot was drawn and spermatozoa were immobilized with 100 μL of a solution containing 0.37% (*v*/*v*) formaldehyde and 0.6 M NaHCO_3_ in distilled water. The immobilized sample was immediately loaded in an A2^TM^ NC-Slide (ChemoMetec, Alleroed, Denmark) and assessed by image cytometry using NucleoCounter^®^ NC-3000^TM^ (ChemoMetec, Alleroed, Denmark). Gates were established that separated viable acrosome-intact (PSA-, PI-), viable acrosome-reacted (PSA+, PI-), and dead spermatozoa (PSA+/-, PI+) based on PSA- and PI-intensity. H342-staining was used to mask objects containing DNA (cells) in the image segmentation. At least 5000 objects or 20 images were analysed per sample. Further details are available in [15,16].

For quality reasons, purified semen samples with a low number of spermatozoa were excluded (*n* = 34, all men from infertile couples). These samples had less than 1500 objects included in the major cell population, which normally appears when analyzing H342 stained sperm cells, on a plot of H342 intensity and H342 area.

The total number of progressive motile spermatozoa with intact acrosomes (TMAI) was calculated by multiplying the acrosomal status with the total number of progressive motile spermatozoa, assuming that only a fraction of the progressive motile spermatozoa has an intact acrosome.

### 2.4. Statistical Analysis

Results were analyzed with the statistical software R, version 3.5.2 (http://cran.r-project.org/). A non-parametric Wilcoxon rank-sum test was used for pairwise comparison of groups and a non-parametric Kruskal-Wallis test was used to test differences when more than two groups were compared. The acrosomal status is not normally distributed [15] and hence we report medians rather than means.

The average deviation from the individual mean across all donors was calculated by subtracting each measurement from the mean of all measurements per donor and taking the average of all donors. The coefficient of variation was calculated as the standard deviation divided by the mean. 

A Pearson’s correlation was applied to analyze the association between frozen-thawed donor sperm samples and pregnancies per sold straw. 

The R package pROC [21] was used for the analysis of receiver operating characteristics (ROC). For analysis of the number of IVF cycles, the data were dichotomized into groups of whether only one cycle of IVF was performed or whether two or more cycles were performed and only measurements with more than 20% viable acrosome-intact spermatozoa were considered. For ROC analysis of pregnancies per sold straw, the data were dichotomized into groups representing the top and bottom decile. A paired ‘roc.test’ using the “delong” method was used to test for differences between ROC curves.

In general, a *p*-value of 0.05 was considered significant. Box Plots depict the median and interquartile ranges, and the whiskers mark 1.5× the interquartile range. 

### 2.5. Ethical Approval

Use of semen from men from infertile couples was approved by the Danish Data Protection Agency (j.no.: 2012-58- 0004) and The National Committee on Health Research Ethics (H-16036581). All sperm donors have given their written consent that their samples can be used for research in accordance with Danish law (LBK 902 23/08/2019 §25).

## 3. Results

### 3.1. Comparison between Sperm Donors, IVF and ICSI Couples

**Figure 1:**
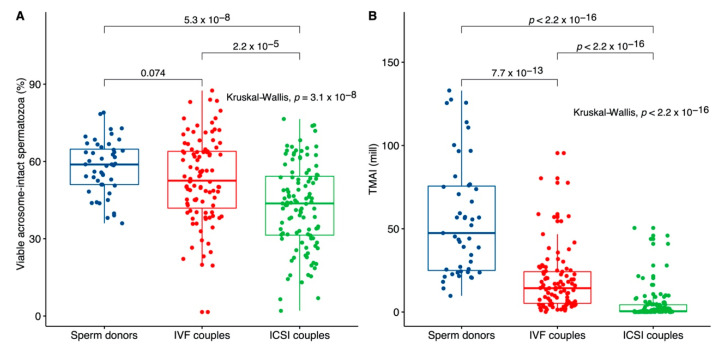
Acrosomal status of sperm donors and couples in in vitro fertilization (IVF) or intracytoplasmic sperm injection (ICSI) treatment. (**A**) Percentage of viable acrosome-intact spermatozoa in fresh purified semen samples, and (**B**) total number of progressive motile spermatozoa with intact acrosomes (TMAI) in fresh purified semen samples (sperm donors (blue): *n* = 48; IVF (red): *n* = 99; ICSI (green): *n* = 107). The boxplots depict the median and interquartile ranges, and the whiskers mark 1.5× the interquartile range.

Table 1 outlines the study populations, their lifestyle factors, standard semen quality parameters, and the acrosomal status in purified semen samples from men in couples undergoing IVF treatment, ICSI treatment and from donors. The fresh purified donor samples had a higher median acrosomal status (58.8%) compared to men from couples in IVF (52.5%) and ICSI (43.7%) treatment (Figure 1A, *p* = 3.1 × 10^−8^). The difference was highly significant between donors and men from ICSI couples (*p* = 5.3 × 10^−8^) and between men from couples in IVF and ICSI treatment (*p* = 2.2 × 10^−5^). This was also significant for the total number of progressive motile spermatozoa with intact acrosomes (TMAI) (Figure 1B). 

ROC curve analysis of acrosomal status with respect to the number of IVF cycles (first vs. second or more cycles) showed a median specificity of 55% and sensitivity of 76% at a threshold of 61% viable acrosome-intact spermatozoa. The area under the curve (AUC) was 65% for viable acrosome-intact, compared to 48% for progressive motile spermatozoa (non-significant; Supplementary Figure S1).

### 3.2. Association with the Primary Cause of Infertility

**Figure 2:**
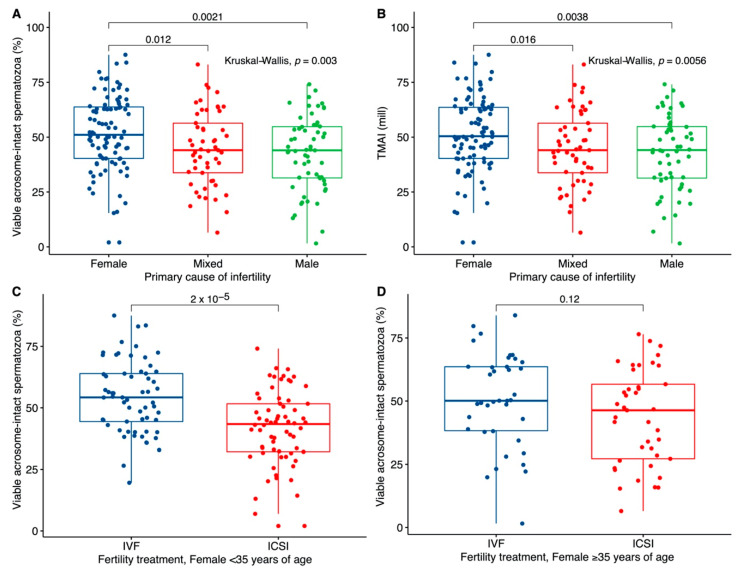
Acrosomal status in relation to the primary cause of infertility and female age. Couples going through fertility treatment were grouped into female, male or mixed causes of infertility. (**A**) Percentage of viable acrosome-intact spermatozoa in fresh purified semen samples versus the primary cause of infertility. (**B**) The total number of progressive motile spermatozoa with intact acrosomes (TMAI) in fresh purified semen samples versus the primary cause of infertility (female causes (blue) *n* = 92, mixed causes (red) *n* = 53, male causes (green) *n* = 59). Percentage of viable acrosome-intact spermatozoa in fresh purified semen samples versus (**C**) the type of fertility treatment for couples with women <35 years of age (IVF (blue): *n* = 61; ICSI (red) *n* = 66) and (**D**) the type of fertility treatment for couples with women ≥35 years of age (IVF (blue) *n* = 38, ICSI (red) *n* = 41). The boxplots depict the median and interquartile ranges, and the whiskers mark 1.5× the interquartile range.

The median acrosomal status of men in couples with primary female infertility was higher (51.1%) than for men in couples with mixed (44.0%) and male (44.0%) causes (Figure 2A, *p* = 0.003). The difference was significant between men from couples with female and mixed causes (*p* = 0.012) and between female and male causes (*p* = 0.0021). A similar difference was observed when TMAI was analyzed (Figure 2B). The statistically significant difference between IVF and ICSI couples (Figure 1) was lost when couples with male or female factors were analyzed alone but remained significant (*p* = 0.0022) for couples with mixed factor infertility. 

In addition, we observed a significant difference between IVF and ICSI men with a female partner below 35 years of age (*p* = 2.0 × 10^−5^), but this became non-significant for men with partners above 35 years of age (*p* = 0.12; Figure 2C,D). There was no significant difference between the couples with female age above or below 35 with regards to the primary cause of infertility but, as expected, a difference in the male partners median age was observed (32 versus 39 years). There was no significant difference in the male age between the IVF and ICSI groups for the couples with a female age below 35 years.

### 3.3. The Influence of Lifestyle and Intra-Individual Variation

**Figure 3:**
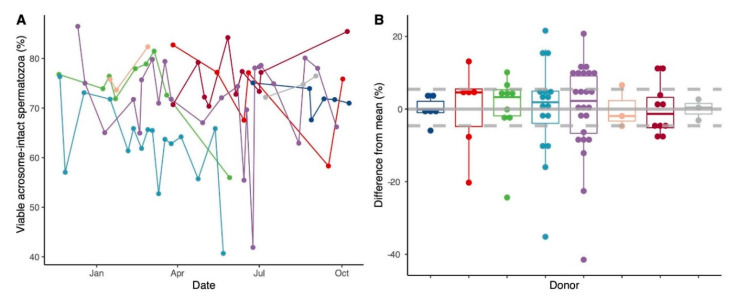
Intra-individual variation of acrosomal status in raw ejaculates of donors analyzed over time (**A**) Acrosomal status in repeated samples over time from eight donors (indicated by eight different colors). (**B**) The intra-individual variation is shown as a deviation (%) from the mean for each donor (depicted by the same colors). The boxplots depict the median and interquartile ranges, and the whiskers mark 1.5× the interquartile range. The average interquartile range of % deviation for all donors (−4.6% to 5.5%) is marked by dashed lines.

We did not observe an association with any lifestyle parameters and acrosomal status. The age of the male partner showed a negative but non-significant correlation to the acrosomal status (Supplementary Figure S2). Further, measurements of individual levels of acrosomal status over time showed a mean individual variation of 44% (range: 5–87%; Figure 3A). The average deviation from the individual mean across all donors was 0.0% (median: 1.18% and range: −41–22%) with an interquartile range of −4.6% to 5.5% (Figure 3B) and a mean coefficient of variation (variation relative to the mean) of 8.5% (range: 2.9–13.5%).

### 3.4. Association with Fecundity

**Figure 4:**
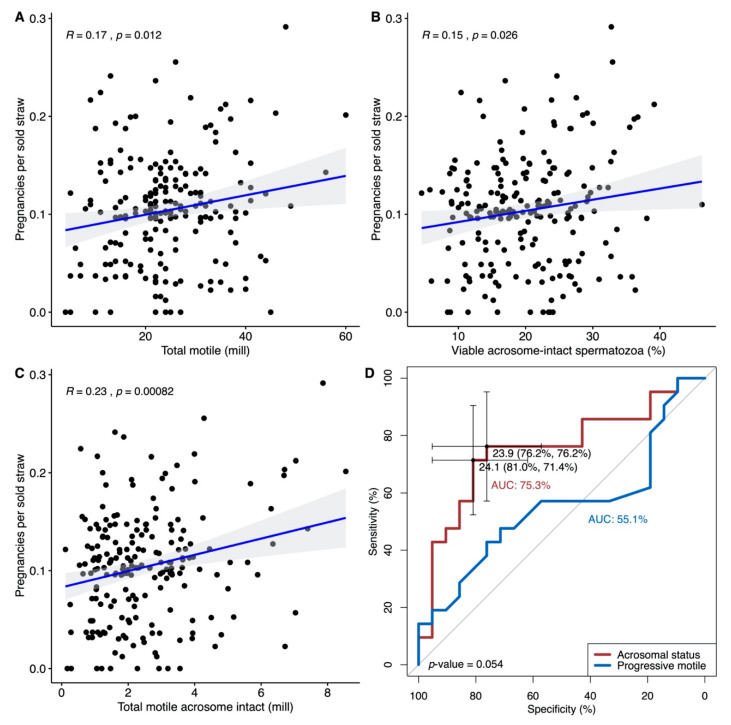
Correlations between frozen-thawed donor sperm samples (*n* = 199) and the number of pregnancies per sold straw: (**A**) concentration of progressive motile spermatozoa, (**B**) percentage of viable acrosome-intact spermatozoa and (**C**) the total number of progressive motile spermatozoa with intact acrosomes (TMAI). The blue line is the best-fitted regression line with 95% confidence intervals in grey and the Pearson correlation coefficient (*R*) and *p*-value (*p*) are indicated in each plot. (**D**) ROC curve analysis of pregnancies per sold straw (top decile pregnancies per sold straw vs. the bottom decile) versus acrosomal status (red line) and progressive motile spermatozoa (blue line) of frozen-thawed samples. The *p*-value indicates the difference between the receiver operating characteristics (ROC) curves.

We observed a positive significant correlation between the number of pregnancies per sold straw (fecundity) and the concentration of progressive motile sperm (r = 0.18, *p* = 0.013, Figure 4A) as well as the percentage of viable acrosome-intact spermatozoa (r = 0.16, *p* = 0.025, Figure 4B). The correlation was also significant for the total number of progressive motile spermatozoa with intact acrosomes (r = 0.23, *p* = 0.0011, Figure 4C).

ROC curve analysis of the acrosomal status of frozen-thawed samples with respect to pregnancies per sold straw (top decile vs. the bottom decile) showed two optimal thresholds with a median specificity of 81/76% and sensitivity of 71/76% at a threshold of 24% viable acrosome-intact spermatozoa. The AUC was 75% for viable acrosome-intact, compared to 55% for progressive motile spermatozoa (the difference between the ROC curves was nearly significant *p* = 0.054; Figure 4D).

## 4. Discussion

This study shows a significant difference in the acrosomal status of samples from men in couples treated by IVF and ICSI even after density gradient purification of the spermatozoa. Our results indicate that the percentage of viable acrosome-intact spermatozoa may be an important parameter when selecting the most appropriate kind of fertility treatment. 

Our study adds information to our previous study [15] on the acrosomal status of raw ejaculates. In raw ejaculates we earlier found that the acrosomal status was lowest in men later undergoing ICSI compared to IUI [15]. In the present study, the semen samples were the purified samples that were used on the same day for either IVF or ICSI treatment. The density gradient purification discards spermatozoa with poor morphology and non-viable spermatozoa [18], which could affect the observed association in the previous study. However, despite the purification, we still observed that the samples used for ICSI treatment had a significantly lower acrosomal status compared to all other groups investigated. Thus, the purification does not appear to affect the relative distribution of spermatozoa with or without an intact acrosome. We did not observe a significant difference among purified samples from sperm donors and couples in IVF treatment. However, this was somewhat expected as couples mainly are referred to IVF due to female factor infertility and thus this group also includes normozoospermic men. Our study included a reasonable number of IVF and ICSI couples but using even larger cohorts could have increased the power of the associations and decreased the influence of confounding factors. The acrosomal status may be less important for ICSI treatments as zona pellucida penetration and membrane fusion are bypassed, but important for IUI/IVF. Especially, couples with no clear male or female factor infertility and several failed IUI/IVF attempts could benefit from early measurement of acrosomal status that could direct these couples to ICSI and in this way advise the physicians at fertility clinics in the choice of optimal treatment of a couple as an add-on to the classical semen parameters.

However, also among sperm donors with good semen quality, we found an association of acrosomal status with the rate of pregnancies per sold straw, indicating that the acrosomal status is important for fecundity. Among sperm donors, both the total number of progressively motile spermatozoa, the acrosomal status and the combination of both (TMAI) showed a significant association with the reported rate of pregnancies per sold straw. ROC curve analysis revealed that acrosomal status is better than the total number of progressive motile spermatozoa in discriminating between the probability of a high number of pregnancies per sold straw compared to a low number. This is in line with other studies that have found that the total number of progressive motile spermatozoa is not a good predictor of donor IUI success [22,23,24]. It can be discussed whether the translation of the number of pregnancies per sold straw into overall fecundity is applicable. Nevertheless, the measurement of acrosomal status may be a relevant future tool for donor choice to optimize treatment success among women requesting sperm donation. However, additional studies are needed to specifically address the added predictive value of the acrosomal status and/or TMAI compared to the currently accepted measure, i.e., the total number of progressively motile spermatozoa. 

Earlier studies on the importance of the acrosome are mainly related to the acrosomal responsiveness, i.e., the ability of the acrosome to react to stimuli, such as an ionophore or progesterone, and release its content by exocytosis [25,26,27,28]. Acrosomal responsiveness is, however, a different matter of concern as it only relates to the proportion of spermatozoa that still retain an intact acrosome. Assessment of acrosomal responsiveness is a time-consuming procedure due to both purification and capacitation steps and therefore not possible to perform in the routine semen laboratory nor in fertility clinics. Furthermore, the acrosome responsiveness has mainly been assessed either by microscopy [7,29,30] or flow cytometry [17,31,32]. The first procedure is laborious and subjective, and the latter procedure depends on the availability of advanced equipment and highly skilled technicians. In contrast, the image cytometer, as applied in the present study, is easy to use because the samples are analyzed in a closed and virtually maintenance free system and requires limited training [15]. 

It is known that chemicals in our environment, like UV-filters, can induce acrosomal exocytosis [25,26]. Such a mode of action may contribute (via the male partner) to the observed decline in fertility which has been described in many countries. Our study population was probably too small to adequately address the question of whether or not lifestyle factors, such as BMI, alcohol and smoking may also have an influence on acrosomal status. Male age has been found to have an effect on some of the classical sperm parameters and sperm DNA fragmentation [27,28,29]; however, we were not able to show a significant correlation between acrosomal status and the age of the male partner, which again may be due to the size of study population. The age of the female partner has a significant impact on the chances of pregnancy and also on the type of fertility treatment chosen [30,31]. Our study showed a difference in acrosomal status between couples in IVF and ICSI treatment when isolating couples in which the female partner was below 35 years of age. This difference was not found for couples in which the female partner was above 35 of age. Other studies have suggested an increased capacity of female oocytes <35 years of age which may compensate for reduced semen quality [32]. Our data is likely to reflect that the treatment choice for infertile women above 35 years of age is more often ICSI than IVF, irrespective of the semen quality of the male partner. However, for the couples in which the female partners were below 35 years of age measurement of acrosomal status may also be an informative parameter in the choice of treatment, potentially avoiding failed IVF attempts and creating an option to further individualize the treatment.

The acrosomal status is likely to be dynamic but repeated measurements over time, somewhat surprisingly, showed a relatively small coefficient of variation indicating a small intra-individual variation. A small intra-individual variation may offer the opportunity to perform the assessment of acrosomal status in the clinical workup of the male partner prior to the day of treatment, where time is limited. In contrast, a small intra-individual variation also implies that there may be limited room for intervention, e.g., to change lifestyle or environmental parameters that negatively influence the acrosomal status. We did nevertheless observe single outlier measurements from several of the donors and a limitation of our study was that repeated analysis was only performed on eight donors. Repeated measurements on more individuals and linkage to changes in lifestyle are needed to establish if associations exist between lifestyle and the acrosomal status, as observed for some of the classical sperm parameters and sperm DNA fragmentation [33,34,35,36].

In conclusion, we observed a statistically significant difference between the acrosomal status of purified semen samples from sperm donors and men from couples in IVF and ICSI treatment as well as between the primary cause of infertility. The individual acrosomal status was relatively stable over time and was associated with fecundity among sperm donors. The acrosomal status could maybe help to give a better picture of male fertility if measured in combination with already established measures. Larger cohorts are needed to validate potential associations of the acrosomal status with lifestyle and environmental factors.

## Figures and Tables

**Table 1:** Study populations.

Group	IVF	ICSI	Sperm Donors	*p*-Value
Samples/men	99/99	107/107	199/138	
Fresh purified samples	99	107	48	
Frozen-thawed samples	-	-	199	
Cycle number	1.0 (1–6)	2.0 (1–6)	Unknown	1.3 × 10^−5^
Infertility cause				
Male Factor	10	49	-	
Female Factor	62	30	-	
Mixed factor	26	27	-	
Unknown	1	1	-	
Lifestyle factors				
Uxor age (years)	33.0 (22–43)	33.0 (24–43)	Unknown	0.492
Vir age (years)	34.0 (23–58)	35.0 (27–57)	26.0 (18–41) ***	0.541
Uxor BMI	22.5 (17.4–34.4) *n* = 82	22.8 (17.3–33.6) *n* = 87	Unknown	0.552
Uxor alcohol (units per week)	0.0 (0.0–6.0) *n* = 75	1.0 (0.0–8.0) *n* = 82	Unknown	0.006
Uxor smoking (cigarettes per day)	0.0 (0.0–10.0) *n* = 77	0.0 (0.0–15.0) *n* = 82	Unknown	0.501
Vir BMI	23.8 (22.0–29.0) *n* = 8	25.7 (22.2–35.2) *n* = 18	23.8 (18.0–37.9) *	0.317
Vir alcohol (units per week)	1.0 (0.0–14.0) *n* = 33	4.5 (0.0–21.0) *n* = 44	Unknown	0.006
Vir smoking (cigarettes per day)	0.0 (0.0–15.0) *n* = 37	0.0 (0.0–17.0) *n* = 45	Unknown	0.041
Semen samples				
Ejaculate volume (mL)	3.0 (0.5–7.5)	3.0 (0.5–7.0)	3.5 (0.7–10.2) ***	0.500
Spermatozoa concentration (mill/mL)	67.0 (10.0–250.0)	15.0 (0.1–118.0) *n* = 106	104.9 (30.6–539.1) *** n = 154	<2.2 × 10^−16^
Progressive motile spermatozoa (mill/mL)	40.0 (4.0–150.0) *n* = 98	5.0 (0.0–50.0)	56.8 (11.3–305.6) *** n = 154	<2.2 × 10^−16^
Percentage viable acrosome-intact after processing (%)	52.5 (1.6–87.5)	43.7 (2.0–76.5)	58.8 (36.0–79.0) *** n = 48	2.2 × 10^−5^

Note: Data are presented as median (range) and *n* = number of data points if different from number indicated in the top row. The *p*-values indicated in the right column compare IVF and ICSI couples with a non-parametric Wilcoxon rank-sum test. All three groups are compared with a non-parametric Kruskal-Wallis test as indicated by an asterisk next to donor data: * *p* ≤ 0.05 and *** *p* ≤ 0.001.

## Supplementary Materials

The following are available online at https://www.mdpi.com/2077-0383/9/8/2327/s1, Figure S1: ROC curve analysis of IVF cycle number versus acrosomal status and progressive motile spermatozoa of purified samples, Figure S2: Correlation between the age of the man and the percentage of acrosome-intact spermatozoa in washed purified semen samples.
