# Supplementary material for: The Acrosomal Status of Density Purified Spermatozoa Differentiates Men from Couples in IVF and ICSI Treatment and Is Associated with Fecundity
Source: J Clin Med. 2020 Jul 22;9(8):2327. doi: 10.3390/jcm9082327 (PMC7464336; doi:10.3390/jcm9082327)
Supplement: Supplementary file 1 [file jcm-09-02327-s001.zip › Supplementary figure_S2_R2.pdf]

Norup et al., The acrosomal status of density purified spermatozoa differentiates men from couples in IVF and ICSI treatment and is associated with fecundity. **Supplementary figure S2**

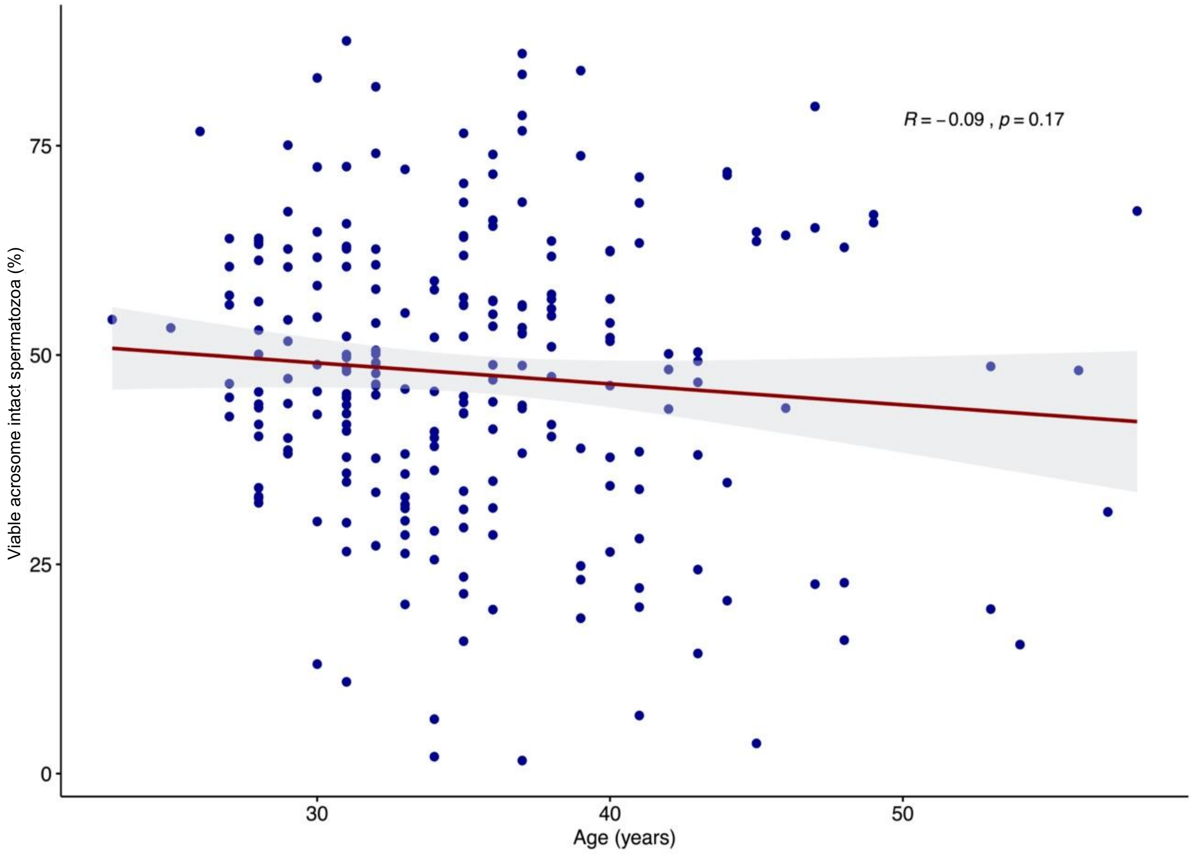

Supplementary figure S2. Correlation between the age of the man and the percentage of viable acrosome intact spermatozoa in washed purified semen samples (n=235). The red line is the Pearson correlation with 95 % confidence intervals in grey.
