# Supplementary material for: The Acrosomal Status of Density Purified Spermatozoa Differentiates Men from Couples in IVF and ICSI Treatment and Is Associated with Fecundity
Source: J Clin Med. 2020 Jul 22;9(8):2327. doi: 10.3390/jcm9082327 (PMC7464336; doi:10.3390/jcm9082327)
Supplement: Supplementary file 1 [file jcm-09-02327-s001.zip › Supplementary figure_S1_R2.pdf]

Norup et al., The acrosomal status of density purified spermatozoa differentiates men from couples in IVF and ICSI treatment and is associated with fecundity. **Supplementary figure S1**

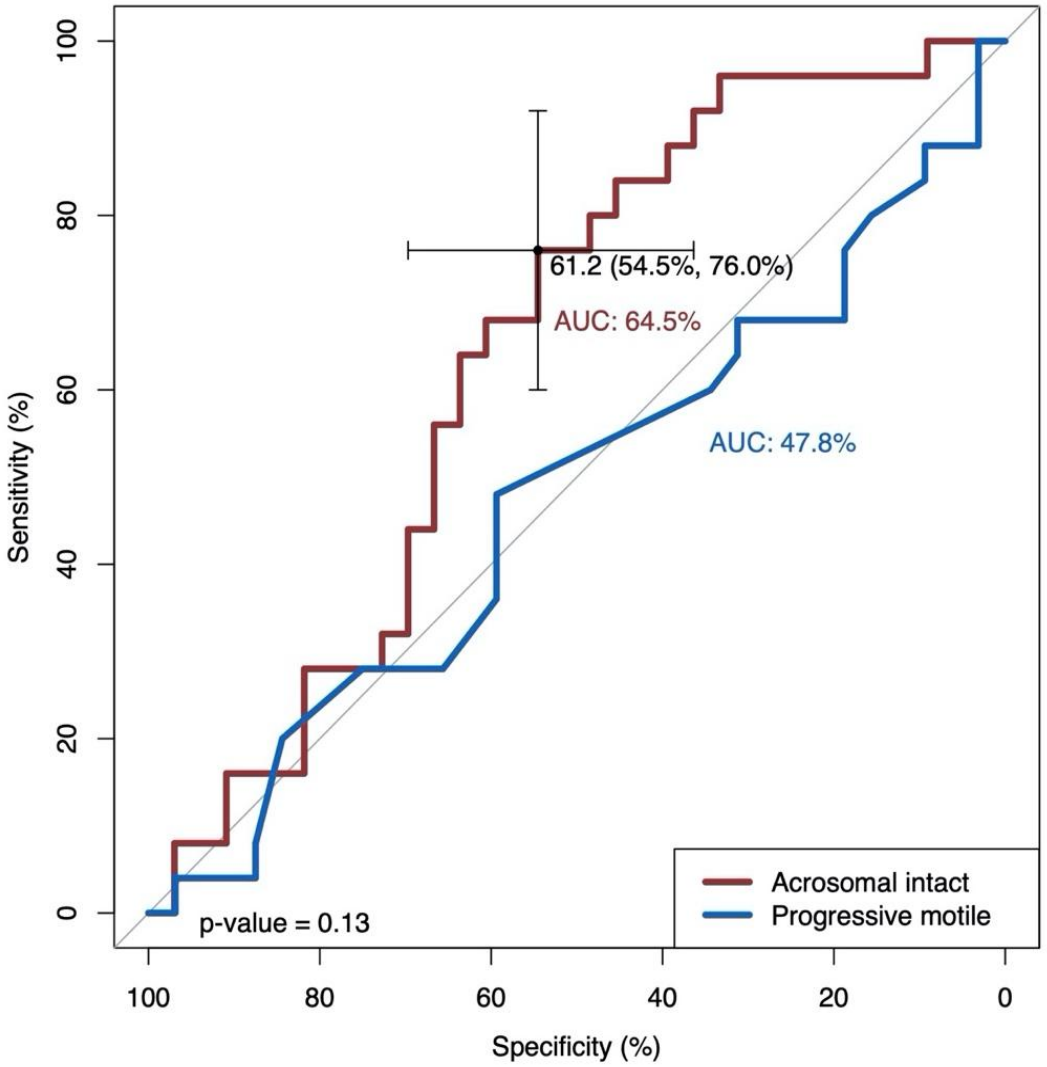

Supplementary figure S1. ROC curve analysis of IVF cycle number (first vs. second or more cycles) versus acrosomal status (red line) and progressive motile spermatozoa (blue line) of purified samples. The p-value indicates the difference between the ROC curves.
